# The impact of a multi-level maternal health programme on facility delivery and capacity for emergency obstetric care in Zambia

**DOI:** 10.1080/17441692.2017.1385824

**Published:** 2017-10-10

**Authors:** Elizabeth G. Henry, Donald M. Thea, Davidson H. Hamer, William DeJong, Kebby Musokotwane, Kenneth Chibwe, Godfrey Biemba, Katherine Semrau

**Affiliations:** a Department of Global Health, Boston University School of Public Health, Boston, MA, USA; b Section of Infectious Diseases, Department of Medicine, Boston University School of Medicine, Boston, MA, USA; c Zambian Center for Applied Health Research and Development (ZCAHRD) Limited, Lusaka, Zambia; d Department of Community Health Sciences, Boston University School of Public Health, Boston, MA, USA; e Ministry of Health, Lusaka, Zambia; f District Medical Office, Kalomo, Zambia; g Ariadne Labs, Boston, MA, USA; h Division of Global Health Equity, Brigham & Women’s Hospital, Boston, MA, USA; i Department of Medicine, Harvard Medical School, Boston, MA, USA

**Keywords:** Health systems, maternal health, impact, evaluation, Zambia

## Abstract

In 2012, *Saving Mothers, Giving Life* (SMGL), a multi-level health systems initiative, launched in Kalomo District, Zambia, to address persistent challenges in reducing maternal mortality. We assessed the impact of the programme from 2012 to 2013 using a quasi-experimental study with both household- and health facility-level data collected before and after implementation in both intervention and comparison areas. A total of 21,680 women and 75 non-hospital health centres were included in the study. Using the difference-in-differences method, multivariate logistic regression, and run charts, rates of facility-based birth (FBB) and delivery with a skilled birth provider were compared between intervention and comparison sites. Facility capacity to provide emergency obstetric and newborn care was also assessed before and during implementation in both study areas. There was a 45% increase in the odds of FBB after the programme was implemented in Kalomo relative to comparison districts, but there was a limited measurable change in supply-side indicators of intrapartum maternity care. Most facility-level changes related to an increase in capacity for newborn care. As SMGL and similar programmes are scaled-up and replicated, our results underscore the need to ensure that the health services supply is in balance with improved demand to achieve maximal reductions in maternal mortality.

## Background

Maternal deaths are highly preventable and yet an estimated 289,000 women die annually worldwide from maternity-related causes (World Health Organization [WHO], 2014). Low-income countries (LIC) disproportionately bear the maternal mortality burden with 99% of all maternal deaths. Sub-Saharan Africa (SSA) alone accounts for more than 60% of the world's maternal deaths (WHO, 2014). Despite steady improvement over the past two decades, Zambia continues to have one of the highest maternal mortality ratios in the world, estimated at 398 per 100,000 live births for 2013–2014 (Central Statistical Office [CSO] Zambia, Ministry of Health [MOH] Zambia, & ICF International, 2014).

Risks of serious complications during childbirth and associated maternal morbidity and mortality can be mitigated by improving access to skilled birth attendants and emergency obstetric care (EmOC) in health facilities (Moyer, Dako-Gyeke, & Adanu, 2013; Paxton, Maine, Freedman, Fry, & Lobis, 2005; Ronsmans et al., 2003). The United Nations has supported two key strategies that target intrapartum-related maternal mortality: (1) universal access to skilled care at the time of childbirth, and (2) ensuring that every woman with complications has timely access to quality EmOC (UNFPA, n.d.). The World Health Organization has echoed support for these strategies with a clear focus on skilled care during pregnancy, delivery, and the immediate postpartum period (WHO, 2006).

One of the biggest challenges in achieving access to skilled care and EmOC in countries with large rural populations such as Zambia is connecting women with obstetric emergencies to necessary care. Cost, distance, and the time required to access care are the primary barriers to using the potentially life-saving intrapartum services that may be provided at health facilities (Bhutta, Darmstadt, Haws, Yakoob, & Lawn, 2009). In 2012 a five-year initiative, *Saving Mothers, Giving Life* (SMGL), was launched in Zambia and Uganda to support evidence-based interventions during labour, delivery, and the immediate postpartum period to reduce the risk of maternal and newborn death. The United States Government's Global Health Initiative implemented SMGL as a public–private partnership with support from the Government of Norway, Merck for Mothers, Every Mother Counts, Project C.U.R.E. (Commission on Urgent Relief and Equipment), and the American College of Obstetricians and Gynecologists. In Zambia, the programme operated in four districts (Lundazi, Nyimba, Kalomo, and Mansa) located in three provinces through the Ministry of Health and the Ministry of Community Development, Mother and Child Health. The programme built on both the Maternal and Newborn Health Roadmap (2007–2014) and the Campaign to Accelerate the Reduction of Maternal Mortality in Africa-Zambia (Zambia Ministry of Health, 2007).

SMGL operated at the community and facility levels to increase demand for, access to, and the quality of obstetric care. SMGL activities were designed to address the three delays that lead to maternal mortality (Thaddeus & Maine, 1994) and included several core components and activities (Table 1). Among these, one of the key activities was to promote facility deliveries and birth preparedness through a cadre of non-clinical, community-based volunteers called Safe Motherhood Action Groups (SMAGs). SMAGs were formed in Zambia in 2003 as part of the Safe Motherhood program and supported initially through the United Nations Population Fund (UNFPA). The groups have since been expanded throughout the country by the Government of Zambia with the training support of several non-governmental organisations (Ensor et al., 2014). SMGL partners expanded the SMAG programme by recruiting additional volunteers and providing training support. Other SMGL activities included developing communications, referral and transport systems for emergencies, and building the capacity of health facilities and their staff to deliver high-quality maternity and newborn care by providing equipment and provider training (Saving Mothers Giving Life, 2014).

**Table 1.** Intervention components and core activities of SMGL as implemented in Kalomo District, Zambia.

*1. Educate and Mobilize Communities to Drive Demand for Facility Delivery*
A. Support and train a cadre of community volunteers as part of Safe Motherhood Action Groups (SMAG) to identify pregnant women in the community and provide messages regarding safe delivery
B. Community sensitisation: deliver key messages about maternity care via radio and through social networks of trained local leaders within the community
*2. Improve Transport and Referral Systems*
A. Improve emergency response referrals via a functional radio system, using an emergency vehicle and community transport systems
B. Refurbish mothers’ shelters as an option for improving access to facilities for women in remote areas
*3. Improve Availability and Quality of Care*
A. Human Resources: hire new clinical staff, train staff in emergency obstetric care and provide ongoing clinical mentorship at facilities
B. Provide necessary equipment and supplies so that staff can provide higher quality care

Our study assessed the impact of SMGL on facility-based birth (FBB), attendance with a skilled birth provider, and facility capacity to deliver emergency obstetric services in Kalomo District in Southern Province, Zambia, during the first phase of implementation from 2012 to 2013.

## Study data and methods

### Study site and data sources

We analysed data from the Zambia Chlorhexidine Application Trial (ZamCAT), a cluster-randomized controlled trial in which 39,797 pregnant women in Southern Province were enrolled and followed through 28 days post-delivery. The goal of ZamCAT was to evaluate the effectiveness of using chlorhexidine cord cleansing to reduce neonatal mortality (Hamer et al., 2015; Semrau et al., 2016). The trial operated in 6 of the 10 districts of Southern Province, including Kalomo District. Within each district, study investigators randomly assigned a total of 90 health facilities (clusters) to either the intervention or control group. Eligible health facilities provided routine antenatal services and had at least 160 births in their catchment area each year. Pregnant women living in the facilities’ catchment areas were identified either at their facility-based antenatal care visits or during community-based antenatal care outreach and offered enrolment.

According to their study group, babies received clean dry cord care (control group) or topical application of a chlorhexidine solution once per day until three days after the baby's cord fell off (intervention group). Field monitors made five home visits to all study participants throughout the course of the study – one antenatal, within two weeks of enrolment, and four postnatal (days 1, 4, 10, and 28 postpartum). All women in the trial, regardless of study group, received a standard package of services that included: a clean delivery kit, referral to a clinic in the presence danger signs for either the mother or baby, and messages about newborn health. Of the 42,356 women screened for the study, a total of 39,679 (90%) participated. Of these, 94% completed the study through the one month postpartum visit. The study team administered a series of questionnaires to the pregnant women before and after delivery that captured both demographics and health behaviours.

The WHO has identified a set of medical interventions that address the direct causes of maternal death, with seven of these interventions defining Basic Emergency Obstetric Care (BEmOC) and an additional two defining Comprehensive Emergency Obstetric Care (CEmOC) (WHO, UNFPA, UNICEF, & AMDD, 2009). Assessment of these core signal functions at the facility level allows for measurement of a facility's capacity to handle obstetric emergencies. As one of these interventions addresses resuscitation of the newborn, this group of seven interventions are also referred to as Basic Emergency Obstetric and Newborn Care (BEmONC) in the literature. For the purpose of our study, we kept the original BEmOC indicators and indicators of emergency newborn care separate.

In ZamCAT, health facility assessments were conducted at facilities where the trial enrolled participants (*n* = 90) in the six districts at both baseline (September–October 2011) and endline (June–August 2013). The health facility assessment tool was based on ‘Monitoring Emergency Obstetric Care: A Handbook’ (WHO et al., 2009) and included an assessment of an expanded set of indicators that covered routine, basic emergency, and comprehensive emergency care for both mothers and newborns (Gabrysch et al., 2012).

Kalomo District was selected by Zambia's Ministry of Health as one of the four intervention districts for SMGL in Zambia and the only one in Southern Province. SMGL activities were launched in Kalomo in early February 2012 with a three-phase rollout, starting with those facilities with the highest volume of deliveries. SMGL was fully operational in all 34 Kalomo rural health facilities by September 2012, nearly 20 months after the ZamCAT was launched. SMGL activities continued to operate for another 13 months through the end of ZamCAT in October 2013. Study investigators collected all facility, household, and individual-level ante- and postpartum data between February 2011 and October 2013.

### Study design

To quantitatively assess the SMGL programme's impact on FBB and delivery with a skilled provider we used a retrospective pre–post non-equivalent comparison group design (Grembowski, 2001). We compared two cohorts of pregnant women from ZamCAT: one that delivered before SMGL was implemented, and one that delivered after SMGL rollout. We treated the women living in Kalomo as the intervention group, while women from three adjacent and socio-demographically similar districts in Southern Province where SMGL was not implemented served as the comparison group. We excluded two of the ZamCAT study districts that we determined to be significantly different from Kalomo in terms of the socio-demographic characteristics of study women.

To examine facility capacity for maternity and newborn care, we also analysed quantitative data from the health facility assessments conducted both before and after SMGL implementation.

### Sample

For the individual-level analysis, the final sample included all women with birth outcome data during the pre- and during-intervention periods in Kalomo (*n* = 6477) and the comparison districts of Choma, Monze, and Mazabuka (*n* = 15,203). For the facility-level analysis, we included all ZamCAT non-hospital health centres in Kalomo (*n* = 22) and the three selected comparison group districts (*n* = 52 at pre and *n* = 53 at post).

### Indicators

The primary outcome of the individual-level analysis was FBB, expressed as a binary variable and reported by the mother in the household survey. The secondary outcome was attendance with a skilled birth provider, defined as a woman's report on the household survey as delivered by a ‘nurse’ or ‘midwife’, and expressed as a binary variable.

The main treatment or independent variable was living in the SMGL intervention district (Kalomo) either before or after the programme was fully operational. Several potential moderating variables were included in the multivariate analysis, including individual-level factors such as age, level of education, literacy level, parity, and number of household members, plus socio-economic status. We used asset ownership as a proxy for socio-economic status by generating scores using an asset index developed through a principal components analysis, modified from the Zambia Demographic and Health Survey wealth index (Rutstein & Johnson, 2004), and indexing the households by score into quartiles.

To measure the facility's capacity to provide emergency obstetric and newborn care services, we used an expanded list of indicators of emergency obstetric and newborn care (Gabrysch et al., 2012) from the health facility assessments. Of the 23 proposed indicators that span multiple dimensions of facility care, we were able to assess 17 as well as all 4 general requirements of the health facility (24/7 service availability, at least 1 skilled provider, communication tools and referral system, and reliable utilities). Table 2 illustrates the 17 indicators we were able to assess in our study, including: all three routine obstetric care indicators, all seven BEmOC signal functions, plus the two additional CEmOC functions, two of three routine newborn care functions, two of seven basic emergency newborn care functions (of which one, resuscitation of a non-breathing baby, is also classified as a BEmOC function), and both of the comprehensive emergency newborn care functions. The proposed indicators not measured in our study include: infection prevention including hygienic cord care (routine newborn care), and antibiotics to mother if the baby was preterm, corticosteroids in preterm labour, alternative feeding if baby unable to breastfeed, injectable antibiotics for neonatal sepsis, and prevention of mother-to-child transmission of HIV (pMTCT) if the mother was HIV positive (basic emergency newborn care).

**Table 2.** Indicators used to identify basic and comprehensive emergency obstetric and newborn care services.

Proposed obstetric and newborn care functions	Assessed in this study
Routine obstetric care	
Monitoring/management of labour – partograph	X
Infection prevention measures for hands	X
Active management of third stage of labour	X
Basic Emergency Obstetric Care (BEmOC)	
Parenteral magnesium sulphate for pre-eclampsia	X
Assisted vaginal delivery	X
Parenteral antibiotics for maternal infection	X
Parenteral oxytocic drugs for haemorrhage	X
Manual removal of placenta – retained placenta	X
Removal of retained products of conception	X
Comprehensive Emergency Obstetric Care (CEmOC)	
Surgery (C-section)	X
Blood transfusion	X
Routine newborn care	
Thermal protection^g^	X
Immediate and exclusive breastfeeding	X
Infection prevention – hygienic cord care	
Basic Emergency Newborn Care (BEmNC)	
Resuscitation of non-breathing baby with bag & mask	X
Antibiotics to mother if preterm/prolonged PROM	
Corticosteroids in preterm labour	
KMC for premature/very small babies	X
Alternative feeding if baby unable to breastfeed	
Injectable antibiotics for neonatal sepsis	
(PMCTC if HIV-positive mother)	
Comprehensive Emergency Newborn Care (CEmNC)	
Intravenous fluids^h^	X
Safe administration of oxygen^i^	X

Notes: List adapted from proposed obstetric and newborn functions (Gabrysch et al., 2012). Existing EmOC signal functions are in italic bold (from the WHO/UN Handbook).

^a^At least one nurse, midwife, general doctor, or OBGYN at facility.

^b^Functioning communication equipment (landline, mobile, or radio). This does not include private cell phones unless the facility reimburses for cost of phone calls.

^c^Facility has a functioning motorised vehicle with fuel that is routinely available and can be used for emergency transportation or access to a vehicle in near proximity that can be used for that purpose.

^d^Facility routinely has electricity for lights and communication (at a minimum) from any power source during normal working hours; there has not been a break in power for more than two hours per day during the past seven days.

^e^The toilet/latrine is classified using criteria: Flush/pour flush to piped sewer system or septic tank or pit latrine; pit latrine (ventilated improved pit or other) with slab; composting toilet.

^f^Improved water source include the following: Piped, public tap, standpipe, tubewell/borehole, protected dug well, protected spring, and rain water.

^g^Thermal protection: drying baby immediately after birth, skin-to-skin contact with mother, wrapping, no bath in first six hours (AMDD, n.d.).

^h^Newborn intravenous fluid kit available in labour ward.

^i^Newborn oxygen available in labour ward.

### Statistical analysis

To assess the SMGL programme's overall impact in Kalomo on FBB and delivery with a skilled attendant, we calculated a difference-in-differences estimate for the two groups. To account for potential bias in this method we also used run charts to both examine the parallel trends assumption and to detect statistically significant shifts in rates of FBB and delivery with a skilled birth provider. Run charts are simple visual analytic tools that are used widely in quality improvement work, including most recently in health care settings, as a way to examine improvements over time (Perla, Provost, & Murray, 2011). The rule used for detecting a shift was at least six contiguous data points above or below the median.

We used multi-level logistic regression to test the net effect of the SMGL intervention by comparing intervention and comparison sites while controlling for potential confounders. We regressed the individual-level outcomes (FBB and delivery with a skilled birth provider) against a dummy interaction variable that we created by taking the product of time (pre-intervention vs. during intervention) by group (SMGL vs. comparison sites).

To account for differences in the Kalomo and comparison site populations, we also matched individuals in the intervention district with a sample of women from the comparison districts who had similar observable characteristics. To do this, we calculated the propensity score for each individual based on the estimated probability that this person might be in the SMGL group (D’Agostino, 1998). We matched women using the socio-demographic characteristics and other predictors that were both different between the two intervention groups and strongly associated with the outcome of FBB in the overall study population. These included: mother's age, mother's tribe, mother's education, parity, distance to a health facility, HIV status, and household asset quartile. Individuals in the comparison areas without near matches were excluded. With that data in hand, we created a comparison group of individuals that did not have exposure to SMGL but shared the same characteristics as the SMGL-exposed group.

To do the matching, we used the ‘greedy 5->1’ algorithm (Parsons, 2001). Next, we measured the average difference in the FBB outcome variable between the participants and the non-participants. We then ran the regression model again with the intervention and propensity score-matched comparison group to estimate programme effects.

For the analysis of the facility-level data, we conducted chi-square tests of significance between the two groups on the proportion of facilities that met the minimum requirement for each of the facility capacity indicators. We used SAS Version 9.3 (SAS Institute, 2011) for all analyses.

The Boston University School of Medicine Internal Review Board and the University of Zambia Research Ethics Committee approved of the protocol and informed consent forms for ZamCAT.

## Results

**Figure 1.**
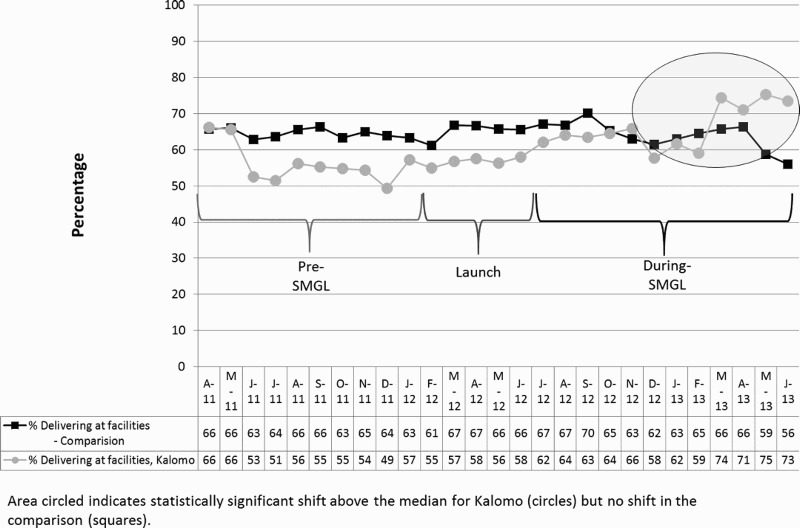
FBB in Kalomo versus Comparison April 2011–June 2013.

The rate of FBB increased from 54.8% in Kalomo before the intervention to 64.6% during the intervention, an absolute difference of 9.8 percentage points (95% CI: 7.4, 12.2, *p* < .01). In the comparison area there was a slight, non-significant increase of 0.2 percentage points (95% CI: −1.4, 1.7). This resulted in a 9.6 net percentage point difference between baseline and endline in the intervention area versus the comparison area (Table 3). Time series analysis revealed that starting in January 2013, there was a statistically significant shift in rates of FBB, with six consecutive points above the median (January 2013 through June 2013). For the comparison group, the time series analysis indicated neither positive trends nor statistically significant shifts during the study period (Figure 1).

**Table 3.** Difference-in-differences analysis of FBB before and during SMGL between SMGL and non-SMGL areas.

Time period	Facility-based birth		Absolute difference (95% CI)
	Pre- SMGL	During SMGL	
Kalomo *N* (%)	54.8 (1567/2859)	64.6** (2336/3618)	+9.8 (7.4, 12.2)
Comparison *N* (%)	64.6 (4881/7559)	64.7 (4949/7644)	+0.2 (−1.4, 1.7)
** **		Net difference	+9.6

**p < .01.

There was 49% relative increase in the odds of FBB in the intervention district after SMGL compared to the comparison area (OR 1.49, 95% CI: 1.21–1.77), adjusting for confounders. There was a significant interaction between area of intervention and time period (*p* = .005) (Table 4).

**Table 4.** FBB before and during the SMGL program implementation time period in Kalomo district and comparison districts.

	Adjusted OR (95% CI)^a^		
	Model 1^b^	Model 2^c^	Model 3^d^
Intervention period × intervention area			
Before, intervention versus comparison	1	1	1
During, intervention versus comparison	1.54 (1.25, 1.83)	1.53 (1.26, 1.82)	1.49 (1.21, 1.77)
Respondent's distance to facility			
<2 h		1	1
≥2 h		0.51 (0.44, 0.60)	0.51 (0.44, 0.61)
Antenatal care			
<4 visits			1
≥4 visits			1.54 (1.35, 1.75)

^a^Consecutive adjustment of covariates in the model and changes of odds ratios of FBB before and during, intervention district versus comparison district.

^b^Adjusted for household size, mother's age, mother's education (any), parity, marital status, and asset quartile.

^c^Adjusted for covariates in model 1 + respondent's distance to facility.

^d^Adjusted for covariates in model 2 + number of antenatal care visits.

Results of the propensity score analysis (PSA) also demonstrated a positive impact in Kalomo. Using multivariate regression with the sample created using PSA, the odds ratio for the difference in FBB between SMGL and non-SMGL groups, before and during SMGL – controlling for household size, maternal age, any maternal education, facility more two than hours away, four or more ANC visits, and parity – was 1.43 (95% CI: 1.14, 1.71). We also observed significant interaction between area of intervention and the time period (*p* = .01).

The proportion of women who delivered with a skilled birth provider increased from 46.2% (1261/2730) in Kalomo before the intervention to 51.7% (1797/3475) during SMGL, an absolute difference of 5.5 percentage points (95% CI: 3.0, 8.0, *p* < .0001). In the comparison area, there was a slight decrease of 0.4 percentage points (95% CI: −2.0, 1.2). This resulted in 5.9 net percentage point difference from baseline to endline in the intervention over the comparison areas. However, using a regression model, the odds ratio for the difference in rate of delivery with a skilled birth provider between SMGL and non-SMGL groups, before and during the programme, controlling for confounders, was 1.27 (95% CI: 0.87, 1.66). There was no significant interaction between area of intervention and time period (*p* = .24).

While were not able to quantitatively assess changes in the overall system of referral, which is essential to improving maternal health outcomes, we were able to assess two of the facility indicators necessary in the referral process: the ability to communicate with the referral centre via radio or phone, and access to transport for referral. In the intervention facilities, there was a statistically significant increase in the proportion of sites with functioning communication equipment, from 4 (18.2%) at baseline to 14 (63.6%) at endline. While there was no significant change from a baseline of 22 (40.0%) to endline of 27 (50.0%) in the comparison facilities, these facilities had a much higher proportion of communications to start with than in the intervention facilities (results not shown). There was not a significant change in the proportion of sites with nearby access to a functioning motorised vehicle that is routinely available for referral in either intervention or control areas, though there was an increase in Kalomo [4 (18.2%) to 9 (40.9%)] and a decrease in the comparison areas [11 (20.0%) to 6 (11.1%)].

The mean number of skilled birth providers at non-hospital health facilities did not change significantly over the study period in either Kalomo (2.50 before to 2.23 during, *p* = .78) or the comparison area (3.36 before to 3.87 during, *p* = .55). In both study areas none of the health facilities met the basic requirements for emergency obstetric and newborn care at baseline, using the seven originally proposed signal functions for BEmOC^1^ (WHO et al., 2009), and there was no detected change over the study period. There were, however, some improvements. The mean number of BEmOC signal functions in Kalomo facilities increased from 2.68 (SD 1.09) to 3.86 (SD 1.39), out of 7, during SMGL compared to before SMGL (*p* = .003) (Table 5), with a non-significant decline in the comparison area. Though not statistically significant, there was an increase from 1 facility (4.6%) at baseline performing assisted vaginal delivery (AVD) to 4 facilities (19.1%) at endline in Kalomo District, with no facilities at either time point performing this important signal function in the comparison area. Of the six newborn care signal indicators that we were able to assess (including basic neonatal resuscitation), four had a statistically significant positive change in Kalomo, compared to only one in the comparison area (Table 5).

**Table 5.** Proportion of non-hospital health facilities with select obstetric and newborn care indicators in Kalomo and comparison districts, before and during SMGL implementation.^a^

	Kalomo		Comparison	
	Before (*n* = 22) *N*(%)	During (*n* = 22) *N*(%)	Before (*n* = 52) *N*(%)	During (*n* = 53) *N*(%)
Routine obstetric care				
Monitoring and management of labour with partograph	18 (81.8)	21 (95.5)	48 (92.3)	48 (90.6)
Infection prevention measures for hands	18 (81.8)	22 (100.0)	50 (96.1)	52 (98.1)
Active management of third stage of labour (AMSTL)	14 (63.7)	19 (90.5)	51 (98.1)	50 (94.3)
Basic emergency obstetric care (BEmOC)				
Parenteral magnesium sulphate for pre-eclampsia	5 (22.7)	6 (27.3)	10 (19.2)	12 (22.6)
Assisted vaginal delivery	1 (4.6)	4 (19.1)	0 (0)	0 (0)
Parenteral antibiotics for maternal infection	20 (95.2)	19 (86.4)	42 (80.8)	29 (54.7)**
Parenteral oxytocic drugs for haemorrhage	20 (95.0)	21 (100.0)	49 (98.0)	50 (96.2)
Manual removal of placenta for retained placenta	6 (27.3)	7 (31.8)	10 (19.6)	12 (22.6)
Removal of retained products of conception	0 (0)	9 (40.9)**	4 (7.7)	2 (3.8)
Routine newborn care				
Thermal protection^b^	2 (9.1)	15 (68.2)**	24 (46.2)	27 (50.9)
Immediate and exclusive breastfeeding	20 (90.9)	20 (95.2)	48 (92.3)	51 (98.1)
Basic emergency newborn care (BEmNC)				
Resuscitation with bag and mask	7 (31.8)	19 (86.4)**	15 (28.9)	17 (32.1)
KMC for premature/very small babies	3 (13.6)	20 (90.9)**	32 (61.5)	46 (86.8)**
Comprehensive emergency newborn care (CEmNC)				
Intravenous fluids^c^	1 (4.6)	10 (45.5)**	11 (21.1)	12 (22.6)
Safe administration of oxygen^d^	1 (4.6)	1 (4.6)	4 (7.7)	3 (5.7)
Mean signal functions for BEmOC^e^	2.68 (1.09)	3.86 (1.39)**	2.51 (1.00)	2.30 (1.39)

^a^List adapted from proposed obstetric and newborn functions (Gabrysch et al., 2012).

^b^Drying baby immediately after birth, skin-to-skin contact with mother, wrapping, no bath in first six hours (AMDD, n.d.).

^c^Newborn intravenous fluid kit available in labour ward.

^d^Newborn oxygen available in labour ward.

^e^Seven BEmOC functions are listed in bold italic.

*p < .05.

**p < .01.

## Discussion

The overall purpose of this study was to assess the impact of the SMGL programme in Kalomo District on both the utilisation of facilities for deliveries and the capacity for intrapartum maternal and newborn care at non-hospital health facilities. We found that SMGL, as implemented in Kalomo, significantly increased the rate of FBB. This was achieved even when taking into account other factors that drive traditional home-based deliveries in Zambia. A previous evaluation of SMGL also showed an increase in facility delivery, from 63% at baseline to 84% at endline across all SMGL districts of Zambia (Centers for Disease Control and Prevention, 2014). However, this evaluation utilised a pre–post with no comparison group design, and collected data at the facility level rather than the household, using population estimates for the denominator when determining rates. Our design provides more robust evidence for this outcome.

One of the key activities in SMGL was recruiting additional volunteers for SMAGs and providing training support in order to communicate key messages about facility delivery. These SMAGs also often escorted women to the local facility when they went into labour. Other studies have found that similar community mobilisation strategies can increase rates of facility delivery. A recent evaluation of a similar programme that utilised SMAGs to raise awareness about maternity care in Zambia also showed a significant increase in facility delivery, from 49% to 75%, compared to the non-intervention areas (Ensor et al., 2014). Similarly, a review of strategies linking families and facilities found that community mobilisation and engagement can significantly increase rates of institutional birth and skilled birth attendance, leading to a reduction in early neonatal mortality (Lee et al., 2009).

Despite the improvement in rates of FBB in our study, there was no significant change in the proportion of women delivering with a skilled birth provider at health centres in Kalomo, though run charts indicate early signs of a shift towards the end of the study period. Importantly, however, the difference between the proportion of women delivering at a facility and proportion of women delivering with a skilled birth provider widened over the course of SMGL's implementation in Kalomo. Before SMGL an estimated 84.3% of women who delivered at facilities did so with a skilled birth provider. During SMGL, this decreased 4.1 percentage points to 80.2%, leaving a small but important difference that highlights the human resources challenges in rural Zambia. A recent analysis of other Zambian-based studies (including ZamCAT) supports this result, finding that nearly 14% of deliveries within health facilities were attended by unskilled staff, the majority of whom (71%) were traditional birth attendants (Biemba, Yeboah-Antwi, Semrau, Hammond, & Hamer, 2014). Another experimental study in Pakistan evaluating a community-based safe motherhood project yielded similar results, with a slight increase in facility delivery but no change in delivery with a skilled birth provider (Midhet & Becker, 2010).

One of the SMGL strategies to improve the rate of deliveries with skilled birth providers in the short-term was to hire and deploy a cadre of retired midwives in areas where they were needed most. Our finding of no change in mean number of skilled birth providers in Kalomo suggests that this strategy was not successful in improving provision of skilled care at non-hospital health centres. One plausible explanation is that midwives were not stationed at the health centres as planned. Discussion with project staff indicated that 6 of these 13 midwives worked in the referral hospitals in Kalomo. Therefore, they could not have been detected in our analysis as they were not at non-hospital health centres. Four midwives worked at four of the 22 Kalomo health centres included in the ZamCAT health facility assessments, but this, representing 18% coverage, was too few to detect a change. The final three midwives worked at two of the nine health centres (22% coverage) that were not in our health facility assessments data set. Even if these additional health centres had been included in our analysis, it is unlikely that there would have been a noticeable change. To create a scenario where there would be a detectable difference in deliveries by skilled birth providers, there must be a strategy to significantly increase the number of skilled birth providers at health centres in a short amount of time, which SMGL seems to have been unable to accomplish.

Another SMGL strategy was formal training of existing health centre staff in Kalomo in emergency obstetric and newborn care as well as a programme called Helping Babies Breathe. In addition, a team of experienced midwives rotated through the health centres monthly providing on-site clinical mentoring to existing staff who cared for women, regardless of their clinical training. Results from a qualitative inquiry conducted by our team indicated that both the trainings and the mentorship visits improved both knowledge of and confidence in providing care in emergency obstetric situations (manuscript under development). There is a body of evidence to support that drills training and supervision can improve knowledge and skill levels of providers (Bailey et al., 2016; Bhutta et al., 2009). In particular, trainings in neonatal resuscitation with providers in LIC have the potential to reduce intrapartum-related neonatal deaths (Wall et al., 2010).

However, a recent evaluation of a skills and drills intervention in secondary-level government facilities in India resulted in an increase in knowledge, competence, and skills but no change in the correct diagnoses and management of maternal or neonatal complications (Varghese et al., 2016). Another long-term study of the effects of in-service training and supportive supervision in seven countries of SSA on antenatal and sick child care indicate overall poor quality of care and low level of implementation of training content (Leslie, Gage, Nsona, Hirschhorn, & Kruk, 2016). While skills and mentorship/supervision may be an important potential strategy in countries with human resource shortages, it is not clear whether this would be sufficient to improve health outcomes without equal emphasis on improving other health systems factors. In India, it was suggested that improvements through skills training and supervision are hampered by insufficient systems-level resources at the facility such as human resources, governance, and supplies (Varghese et al., 2016).

A recent review reported that while demand-side interventions for improving maternity care have been shown to improve utilisation of facilities, there is a weak evidence for improvement of the health outcomes (Hurst, Semrau, Patna, Gawande, & Hirschhorn, 2015). Our repeated health facility assessments showed only a slight increase (just over one) in the average number of signal functions performed at health centres during SMGL implementation. There was a statistically significant, positive change in the proportion of facilities performing the removal of retained products of conception from baseline to endline. Although not significant, there was an increase in the proportion of facilities that reported performing AVD. While both improvements are important, AVD is a critical signal function that can directly improve both maternal and neonatal outcomes, particularly in low-resource settings where access to Caesarean sections may be limited (Bailey et al., 2017). In Zambia, where only between 1% and 4% of non-hospital health centres practice AVD (Bailey et al., 2017; Owens et al., 2015), and only 6% of health centre staff have been reported to be comfortable with the skill (Levine, Marsh, Nelson, Tyer-Viola, & Burke, 2008), the positive change found in our study warrants recognition. It also highlights the importance of employing a comprehensive package of interventions to address supply-side challenges in this context in order to change health outcomes, especially activities relating to clinical trainings, skills transfer, and mentorship.

Changes in indicators of facility capacity, mostly in newborn care, can be linked to the content of the emergency obstetric and newborn care and Helping Babies Breathe trainings. This is consistent with an external process evaluation of SMGL that found improved knowledge among providers trained by the project in emergency obstetric and newborn care (Kruk et al., 2016). Taken together, these results indicate a modest improvement in supply-side indicators of care, but also demonstrate insufficient progress that may limit the impact on health outcomes.

Women's cultural beliefs and perceptions of facility-based care have also been found to be major drivers of facility delivery (Moyer et al., 2013). The relatively small change in supply-side factors may have also minimised the observed increase in rate of FBB in Kalomo. Perceived quality of the facility can deter women and encourage them to bypass primary care facilities in favour of referral hospitals (Kruk et al., 2009) or choose to deliver at home (Sialubanje, Massar, Hamer, & Ruiter, 2014). Several recent studies indicate that staff shortages, poor quality of EmOC services, and previous experience of disrespect and/or abuse discourages facility deliveries (Dogba & Fournier, 2009; Larson, Hermosilla, Kimweri, Mbaruku, & Kruk, 2014). While the present data do not allow us to assess the change in FBB over a longer period, it is conceivable that if supply-side factors do not improve, the gain in FBB due to SMGL's success with demand creation could reverse.

Our study exploited the fortunate timing of a large-scale RCT that was launched before programme implementation in the SMGL intervention district, plus comparison districts in Southern Province in which SMGL was not operating. Utilising existing data and designing a quasi-experimental study with imperfect controls can nevertheless strengthen conclusions drawn about a programme even when an RCT is not feasible. Moreover, our combination of several traditional analyses, including difference-in-differences, multi-logistic regression, and time series analyses strengthen the evidence upon which these conclusions are drawn. This study contributes to the growing use of non-traditional, real-world approaches in implementation science and evaluation.

Despite this unique approach used for evaluation, we were limited in our use of existing ZamCAT data, including the selection criteria for the study. Therefore, we can neither generalise to all facilities nor to the patient population of the four districts since the characteristics of the facilities and the women served by the non-eligible facilities may be different. In addition, the women surveyed had to be willing to participate in a newborn health research study. However, since only 10% of the women screened were not enrolled, and only 3.2% were lost to follow-up, it is highly unlikely that refusal to participate was a significant source of bias. There is also potential bias in women's self-report of their facility delivery, especially since both ZamCAT and SMGL included messaging about the importance of delivering at a facility. To help mitigate this, the woman was asked to confirm the specific location of her delivery, not just whether it was in a facility or not.

The recruitment criteria for the ZamCAT omitted women who neither attended antenatal care nor participated in community-based antenatal care outreach activities. It is conceivable, therefore, that the true rate of FBB could be lower than what was reported since those women not attending antenatal care would be more likely to have delivered at home and would have been included in the denominator when calculating the FBB rate. However, given the high antenatal care coverage in Southern Province, estimated at 97.7% in 2013–2014 (CSO Zambia et al., 2014), it is likely that the study sample is in fact representative of most pregnant women in the selected districts.

The activities that took place as part of the ZamCAT may have influenced the outcomes, especially counselling provided by field monitors about facility-based care seeking and delivery. This would have biased our findings towards the null. However, we saw no change in the comparison areas where women got the same messaging from field monitors but not the SMGL intervention.

Differences between individuals living in Kalomo and comparison districts limit their comparability. We attempted to address this with a PSA (D’Agostino, 1998) and our results still showed an impact on facility delivery. However, the ministries selected Kalomo District for SMGL implementation due to several factors, including the strength of local leadership and its motivation to cooperate, and this may have influenced the change in outcomes. These outcomes are also not generalisable to the other four learning phase districts in Zambia as implementation varied.

## Conclusion

SMGL had a major impact on improving the rate of facility-based delivery, and more women accessed health centres to deliver babies with the programme's implementation in Kalomo. However, the gap between increased FBB and the non-commensurate level of provision of high-quality maternity care at Kalomo's health centres suggests that the SMGL model, as implemented in that district, was more successful at creating demand than improving supply. As SMGL and other similar programmes are scaled-up and replicated both nationally and in the Sub-Saharan African region, our findings underscore the need to ensure that the health services supply is in balance with improved demand to achieve maximal reductions in maternal mortality.

## Note

1 The seven BEmOC signal functions include: (1) Administer parenteral antibiotics, (2) Administer uterotonic drugs, (3) Administer parenteral anticonvulsants for pre-eclampsia and eclampsia, (4) Manually remove the placenta, (5) Remove retained products of conception, (6) Perform AVD (e.g., vacuum extraction, forceps delivery), and (7) Perform basic neonatal resuscitation.
